# Bergman Versus Volker

**Published:** 1876-11

**Authors:** Charles C. F. Gay

**Affiliations:** Buffalo, N. Y.


### Correspondence.

### BERGMAN VS. "DR." VOLKER.

### "Suit for Malpractice involving the Surgery of the Hip-Joint."

### A REPLY.

*Editor Buffalo Medical and Surgical Journal:*

Dear Sir.—The October number of the Journal is received, in which is published the medical testimony in the "Suit for Malpractice involving the Surgery of the Hip-Joint, condensed from the notes of the Superior Court Stenographer," with editorial remarks.

Comparing my own testimony published in the Journal, with the Court Stenographer's report—a copy of which I have before me—I find several errors, but in the main, the report, what there is of it, is sufficiently correct to serve the purpose of its publication.

On page 92 the fractional figure $\frac{3}{4}$ is changed to $2\frac{3}{4}$. On the same page the word "Eczematous" occurs in place of Scales; the first two lines on page 93 is an interpolation, to the doctrine of which I do not subscribe. Errors occur also in the testimony of the other medical witnesses which I will not notice.

In two or three paragraphs on page 118 of your editorial, you make such a grave arraignment that your readers will expect to know what the undersigned has to say in his own behalf. You say, "it is certainly a very serious charge for him to say that four men of large experience &c.," "would deliberately perjure themselves to detract a certain amount of merit," &c. I suspect you must have drawn upon your imagination when you indicted that sentence. Perhaps some evil spirit suggested the thought to you. However I must plead innocent to the charge. I certainly cannot allow you the privilege, if it be such, of placing me in any relation with these gentlemen whereby I am made to offer any such affront as you charge. Allow me to rehearse the leading facts of the case under consideration.

On August 31st, 1875, Bergman was knocked down in the street by a passing vehicle. He was struck upon his right side. How he fell to the ground is not known. The blow was certainly not direct. He has large muscular development, and therefore much more liable to dislocation from an indirect blow than a lean man of the same age. He is helped up, puts his hand upon his left hip and complains of great pain. "Dr."? Volker was called upon to render professional services, but after making a few visits retired from the case on the ground that he could not give the patient any relief. On December 11th, 1875, he first came under the observation of Dr. J. F. Miner, and subsequently Dr. Hineman, a homœopathic physician, "commenced" as he testifies "to treat him first in February, 1876." On May 18th, 1876, Dr. Loomis invited the writer to visit the patient along with him. Dr. Loomis had not yet seen Bergman, but it was represented to him that the sick man had swollen limbs. Anticipating a case of œdema of the lower extremities, or perhaps of Elephantiasis, Dr. Loomis requested me to accompany him that we might together be able to testify to the patient's condition in a suit—not the recent one—then in contemplation.

We found considerable, but not excessive œdema of the lower limbs, which were also covered with scaly sores. A thorough examination was instituted to determine whether there was any organic disease—as of the heart, liver or kidneys—which would give rise to the existing œdema. No such disease was discoverable. Our attention was then directed to the left leg of the patient, where were found all the symptoms which Sir Astley Cooper, Hamilton and Malgaigne describe as indicative of dislocation of the head of the femur upon the dorsum of the ilium; in other words we found the left leg shortened over two inches, the leg and thigh inverted and the toes lying over upon the dorsum of the opposite foot. The limb resisted all attempts at eversion. There was a bony prominence upon the dorsum of the ilium; so well marked were these symptoms we had no difficulty whatever in agreeing upon a diagnosis, and either of us would have been willing to go into court the next day and swear to the fact of existing dislocation.

Eight days later, Bergman was put under chloroform and examined by the following physicians; Drs. Burwell, Hauenstein, Hopkins, Diehl, Bartow and myself, six in all. All of these gentlemen were cognizant of the above mentioned symptoms. They were witnesses of the fact that the limb could not be enverted beyond the perpendicular without turning the patient's entire body forcibly over. I at first manipulated the limb to break up the adhesions; this achieved, I rotated the thigh inwards and turned the head of the bone out, placed my right hand upon it and felt it to be unmistakably the head. Dr. Burwell was then invited to place his hand upon it which he did; as did also Drs. Hopkins, Bartow and Diehl. They placed their hands upon the head of the femur and recognized what they felt to be the head of the femur. Dr. Hauenstien was engaged in giving chloroform, and I am unable to state whether or no he placed his hand there.

There could be no mistake here as to the fact. Not one of the physicians made any doubt at the time, that the hip-bone was out of its socket. The evidence adduced is, as you will observe the best possible evidence at the Surgeon's command, viz: the direct demonstrative evidence of the senses; and the force of it is augmented to the highest degree by the concurrent touch of five physicians.

I then resumed manipulations for the purpose of reducing the luxation, after frequent failures I finally succeeded. I felt the head of the bone slip into the acetabulum as I supposed. All signs of dislocation now disappeared. The freest eversion became at once possible. There was no shortening perceptible, for the time being.

Well, what was the upshot of it all? As a matter of course Bergman suffered a few days from the prolonged use of the chloroform, from the breaking up of the old adhesions, and the repeated manipulations; but at the end of seventeen days the dressings were removed and the man was allowed to go about with the aid of crutches. His pains had vanished, and the œdema, the ulcers and scales rapidly disappeared from his legs.

Drs. Loomis, Hauenstein, Hopkins and Bartow, were at one time present in the court-room during the recent trial to corroborate my testimony in the above particulars. But inasmuch as the plaintiff's counsel rested the case on my testimony, they were ruled out on the ground that to take their evidence on this point would be a reopening of the case. It is accordingly obvious that the test-facts in the whole business were not developed in all their force by the late contest at law.

On October 13th, four and a half months after the dislocation was reduced, a commission of experts, consisting of Drs. Moore, White, Miner and Rochester, was appointed by the court to examine the man Bergman. These gentlemen proceeded to measure Bergman's legs, and their measurements varied, as I have testified from $\frac{3}{4}$ of an inch to $2\frac{5}{8}$ inches; these figures were furnished me by Dr. Nichell; so that the first measurement of Dr. White differs from the last measurement of Dr. Miner by *one inch and seven-eighths.* These gentlemen however, finally agreed upon *two* inches as the amount of actual shortening. This discrepancy between their successive measurements is not so ludricous as it might at first seem; on the contrary, it possesses considerable medico-legal importance, and I therefore call attention to it.

The existing shortening is from the most careful measurement the writer can make, 1½ inches at the most. It will fall below this by half an inch, rather than exceed it by any fractional part of an inch. To be surgically accurate, I would add that when this man sets, lies or stands, shortening is scarcely discernible; i. e. when the heels are brought together they show nearly or quite equal length of limbs. Dr. Moore made another important measurement, viz: that "from the major trochanter to the superior spinous process of the ilium." The distance on the injured side was found to be ½ inch shorter than upon the other. The patient was also "made to walk across the room;" a feat he would have hardly accomplished previous to the reduction of the bone.

In what position, then, is the commission of experts. They are simply in possession of two facts of significance; first, that there are 2 (?) inches shortening of Bergman's left leg; second, that the trochanter is ½ inch nearer the anterior superior spine of the ilium than it should be. This is the sum-total of their knowledge as acquired by actual observation. They can go back from the present state of things to a past one only by inferences, by probable reasonings. And I beg leave to insist upon this contrast between the two kinds of evidence presented. The writer and those physicians present at the reduction of the bone, speak with certainty of a luxation, because they had the *demonstration* of it; they handled the dislocated head of the femur. The commission can only supply *inferences,* can only state what was the probable or improbable condition of the joint during the period which followed the injury. For although Dr. Miner had seen the case prior to October 13th, he himself testifies that he had never previously examined the joint for either fracture or dislocation.

But what theory of the case does the commission of experts propose? It reads thus; "there was no dislocation, but on the contrary an *impacted intra-capsular fracture*, which at an early date before separation of the impacted fragments and shortening took place might have been easily overlooked," and it is added that "the treatment adopted by the defendent, in a man of Bergman's age, viz: rest in bed and liniments, was as good as any." In other words, Bergman lay in bed from August 31st, 1875, to May 26th, 1876, nearly *nine* months with an impacted intra-capsula fracture, under a treatment for the first week that was "as good as any" and with no treatment especially differing from the same (so far as the joint was concerned) during the remainder ot the period of nine months without amendment, nay, suffering great pain and almost rotting to death, but when (mark the contrast) a severe operation for the reduction of a dislocated bone—a *wrong* operation—was performed on him May 26th, his pains cease, his bloated extremities resume a normal size, the sores upon them begin to heal and his health improves steadily, all during the space of seventeen days. If such results come of a *wrong* treatment, what patient would prefer a *right* one? Were results alone to be considered, the theory of the commission of experts might be promptly pronounced weak and untenable.

Dr. Miner testifies as follows: "When the neck was fractured within the capsule *some surgeons* formerly claimed that no bony union took place. * * * In *numerous* cases I think it is united by *bone*, and when united, it is just as perfect" &c. Again, "the neck *unites by bone* or ligament in *nearly* its old position" &c. Did Dr. Miner really mean the Jury to understand that in the case of Bergman—61 years old—an intra-capsular fracture would within any range of likelihood unite by *bone*, and leave such a perfect condition of things as he describes? If Bergman is too old for dislocation, surely he is too old for such a reparative feat as that. Such suggestions may be palmed off on a non-medical jury, but hardly upon a physician of tolerable information. But, after all, *is* the present status of Bergman's hip-joint inexplicable on the supposition that the bone was reduced by me on the 26th of May last? Before answering in detail, allow me to notice a sentence I have come across on your editorial page; "Dr. Gay now candidly says that he did not reduce the dislocation." Nothing could leave a falser impression behind it than such a statement. I affirm most positively that on the 18th of May, I found the head of the femur upon the dorsum ilii, that on the 26th, I manipulated to reduce the bone, and that in virtue of my efforts on that day the head of the femur was removed from the dorsum. At the time I felt confident that it had gone into the acetabulum. Subsequently I began to fear that I had been less successful than I hoped, and that the head might have gone into the ischiatic notch. It is well known that such an event may befall any surgeon, and that the operator should be on his guard to forestall it. I affirm that at present the bone is either in its proper socket or in the ischiatic notch. Even if it be in the latter, the operation has been an unmixed benefit to Bergman, since the bone will in time there create for itself a good joint, indeed the "mobility of the leg and the smoothness of the joint surface," which Dr Miner found, would argue that such a joint is already formed. 'Tis of no great account for the question at issue, whether the head be now in the acetabulum or in the notch. That question is this. Was there a fracture or a dislocation? It is quite as subversive of the theory that there was no dislocation to have removed the head of the femur from the dorsum ilii into the ischiatic notch as into the acetabulum. One of these two things I have certainly done. I have therefore disproved any such theory.

And now let me answer the question previously propounded.

First.—Can the present status of the hip-joint be explained on the hypothesis, that the head was reduced into the acetabulum?

Second.—Can it be explained on the theory that it passed into the ischiatic notch?

If no shortening of limb had occurred, it is safe to say there would have arisen no disputation as to the fact of dislocation.

No attempt was made at the recent trial to account for shortening on the hypothesis of reduction into the acetabulum, and since discussion of this question would make this communication of too great length I defer it for a separate article, and proceed at once to answer the second question propounded above, viz:

Is the head of the femur in the ischiatic notch?

It is the unanimous testimony of a majority of the expert commission that the head of the femur is now in the acetabulum and not in the ischiatic notch. This opinion they base upon the fact of perfect "mobility" of the limb and "smoothness of the joint-surfaces" etc. But it must be remembered these gentlemen never examined this man with a view to ascertain whether the head of the femur was lodged in the notch or not. It is fair to presume—since they were so intent upon obtaining all the shortening possible,—that they never thought of examining the limb in order to ascertain if the head was in the notch.

Now what are the signs and symptoms denoting ischiatic displacement. I find the following, viz: 1st, shortening; 2d, Syme's curves; 3d, ¾ of an inch approximation of the trochanter major to the mesial line of the spine upon the left side, with ½ inch approximation of "the trochanter to anterior spine, according to Moore's measurement. Mr. Syme has called attention (Hamilton) to what he considers as one of the most important diagnostic marks; indeed, he says it is never absent, nor is it ever met with in any other injury of the hip-joint, whether dislocation, fracture or bruise; this is the lumber arch."

I can do no more, at present, than call attention to these signs, leaving to the decision of your readers the question, whether or not they should be disrespected.

When the results of the treatment of this interesting surgical case are considered, I have no fear whatever of detraction "from him (myself) of a certain amount of merit," &c., * * whether the head of the femur be lodged in the acetabulum or ischiatic notch. The merit, should any be accorded, I think will consist in having discovered an ancient dislocation upon the dorsum ilii, and in having promptly reduced the head of the bone from the dorsum, if not into the acetabulum—into the ischiatic notch where the joint seems almost as perfect as before the injury.

Reciprocating fully, Mr. Editor, your expressions of kindly personal regards, I am, Truly yours,

Buffalo, N. Y, CHARLES C. F. GAY.

